# Repeatability, reproducibility and interocular difference in the assessments of optic nerve OCT in children– a Swedish population-based study

**DOI:** 10.1186/s12886-018-0940-x

**Published:** 2018-10-22

**Authors:** Eva Larsson, Anna Molnar, Gerd Holmström

**Affiliations:** Department of Neuroscience/Ophthalmology, Uppsala University, SE-751 85 Uppsala, Sweden

**Keywords:** Optical coherence tomography (OCT), Children, Normal values, Repeatability, Reproducibility, Interocular difference

## Abstract

**Background:**

The aim was, first, to collect normative data of the optic nerve head and the peripapillary retinal nerve fibre layer (RNFL) thickness assessed with Cirrus SD-OCT, in healthy children in a population-based study; second, using these data, to examine repeatability, reproducibility and the interocular difference.

**Methods:**

One-hundred and ten eyes from 57 children aged 6–15 born at term, were examined. Best-corrected visual acuity and refraction were assessed. Both eyes were examined and the interocular difference was calculated. Repeatability was calculated by one examiner performing three assessments. Thereafter, a second examiner repeated the assessments to calculate reproducibility.

**Results:**

Mean RNFL thickness was 99.2 (SD 8.8) μm, mean disc area 1.89 (SD 0.37) mm^2^ and mean rim area 1.52 (SD 0.26) mm^2^. No significant correlations with age, gender or refraction were found. Repeatability and reproducibility were good overall. There was interocular symmetry between the eyes.

**Conclusions:**

Normal values for optic nerve head and RNFL thickness assessed with Cirrus SD-OCT were gathered to obtain a normal material in children. High repeatability and reproducibility indicated reliability of assessments performed by different examiners on different occasions. Overall, good correlation between right and left eyes was found.

## Background

Optical coherence tomography (OCT) was first introduced in 1995 [1]. This fast, non-invasive technique has been found to be useful in the investigation of children with various macular pathologies [2, 3]. It has also become an important tool for diagnosing and monitoring optic nerve diseases, such as optic nerve tumours, idiopathic intracranial hypertension and glaucoma [4]. Normal values are necessary to interpret the findings, but for children they are not provided by the OCT software. To date, several OCT studies have been performed regarding normal values in children [5–8], but only a few authors have studied repeatability and reproducibility in children [9]. Similarly, there are few studies of interocular differences in the RNFL thickness and optic nerve head in children [8, 10, 11], although these are important in evaluation of monocular diseases.

The primary aim of this study was to collect normative data on optic nerve head and peripapillary retinal nerve fibre (RNFL) thickness, assessed with Cirrus SD-OCT, in a population-based cohort of full-term healthy children. A second aim was to examine intra- and inter-observer variability and the interocular difference in the Cirrus OCT assessments.

## Methods

Children aged 6–15 years, born at term (≥ 37 weeks of gestation), at normal birth weights (≥ 2500 g), living in Uppsala County were randomly chosen from the birth register of the Swedish National Board of Health and Social Welfare. Ethical approval for the study was obtained from the Regional Ethical Review Board in Uppsala, Sweden. Where applicable, both caregivers of each participating child had to provide written consent.

Assessments were performed at the Department of Ophthalmology, Uppsala University Hospital, Sweden, between September 2012 and December 2013. The inclusion criteria were normal health and no eye disease, best-corrected visual acuity of at least 0.1 logMar, spherical equivalent between + 3.0 and − 3.0 dioptres (D) and astigmatism less than 2.0 D.

Monocular visual acuity (VA) was assessed using linear logMAR charts. If the child was unable to read, an HVOT chart read at 3 m was used [12]. Autorefraction during cycloplegia was performed after dilating the pupils with a mixture of phenylephrine 1.5% and cyclopentholate 0.85%. The fundus was then examined.

The optic disc and the peripapillary retinal nerve fibre layer were measured with spectral domain Cirrus, version 6.0.2.81 (Carl Zeiss Meditec Inc., Dublin, CA) by using the optic disc cube 200 × 200 protocol. The scans were performed through dilated pupils by two examiners. Internal fixation was used. First, three assessments of the optic disc were performed by one of the authors (AM) and the assessments were then repeated by another experienced examiner. The right eye was assessed first by both examiners. The disc size measurements were automatically done by the OCT machine. Peripapillary RNFL thickness was recorded in four sectors (superior, temporal, inferior and nasal) around the optic disc and as an average value. Disc area, rim area, average disc/cup ratio, vertical disc/cup ratio and cup volume were also assessed. The inclusion criteria for the scans were a signal strength of 7 or more and the image being centred on the optic disc. There had to be no eye movement and no blink over the measured area and the thickness boarders automatically drawn by the algorithm of the Cirrus machine were visually validated.

### Statistical methods

Statistical analyses were performed using SPSS version 21. Two examiners performed three measurements on each child. The mean values of the three measurements for each examiner were calculated. The descriptive values were based on the assessments of one of the examiners (AM). The Kolmogorov–Smirnov test was used for analysing normal distributions. Correlations between optic nerve parameters and VA, age and refraction were performed using Pearson’s correlation test. The independent sample T-test was used for analysing differences between boys and girls. To avoid overestimation one eye of each child was randomly chosen when comparing the optic nerve with age and gender. The randomisation was performed with the Excel, Microsoft Software. Repeatability (intra-observer variability) was calculated using the three measurements performed by the first examiner. Reproducibility (inter-observer variability) was calculated by comparing the mean values of the two examiners. Repeatability and reproducibility were expressed as a coefficient of variance (CV), i.e. the standard deviation divided by the mean, and an intraclass correlation (ICC). A CV close to 0 and an ICC close to 1.0 are regarded as perfect. Repeatability and reproducibility were also illustrated by Bland–Altman plots [13]. Interocular difference was analysed in children in whom both eyes could be examined. The difference was analysed using Wilcoxon signed rank test. and the correlation using Pearson’s correlation test. The mean difference between the eyes was calculated and the limits of agreement (±1.96 SD) noted.

## Results

Fifty-seven children, all Caucasian, of those who accepted the invitation (58) were able to complete the assessments in at least one eye. No child was excluded due to low vision or high refractive errors. Of the 57 children, 28 were girls. The mean age of the children was 10.7 years (SD 2.8, range 6–15). In four children, only one eye could be assessed and consequently 110 eyes were analysed. The mean value of VA in the right eyes (RE) was − 0.04 logMAR (SD 0.09, range − 0.2–0.1) and in the left eyes (LE) –0.05 logMAR (SD 0.07, range − 0.2–0.1) and the mean spherical equivalents were 0.95 (SD 0.63, range − 0.5–2.75) and 0.93 (SD 0.71, range − 0.75–3.0) in the RE and LE respectively. No statistical difference was found between RE and LE regarding VA and spherical equivalent.

Mean values of the optic disc measurements, average RNFL thickness and the RNFL thickness in the superior, temporal, inferior and nasal sectors are given in Table 1. All values, except cup volume, were normally distributed.

**Table 1:** Mean values (SD) and ranges of optic nerve head and peripapillary retinal nerve fibre layer (RNFL) assessed with Cirrus OCT, with intra- and inter-examiner variation

	First examiner (*n* = 110)		Intra-examiner variation (*n* = 105)		Inter-examiner variation (*n* = 108)	
	Mean (SD)	Range	CV mean (SD)	ICC (CI)	CV mean (SD)	ICC (CI)
Average RFNL thickness (μm)	99.2 (8.8)	75.3–122.5	1.6% (1.2)	0.943 (0.920–0.961)	1.5% (1.4)	0.940 (0.914–0.959)
Inferior	130.8 (15.1)	91.3–168.5	2.9% (2.1)	0.916 (0.882–0.942)	2.8% (2.4)	0.889 (0.841–0.923)
Superior	123.2 (14.8)	66.0–167.0	3.0% (2.6)	0.848 (0.791–0.893)	2.8% (4.1)	0.851 (0.790–0.896)
Nasal	74.4 (11.5)	47.0–102.3	3.3% (2.4)	0.925 (0.895–0.948)	3.1% (2.6)	0.925 (0.892–0.948)
Temporal	67.8 (8.2)	47.3–84.0	3.9% (11)	0.894 (0.853–0.926)	3.2% (4.8)	0.767 (0.677–0.835)
Disc area (mm^2^)	1.89 (0.37)	1.09–3.02	4.2% (5.4)	0.919 (0.887–0.944)	2.9% (4.1)	0.949 (0.926–0.965)
Rim area (mm^2^)	1.52 (0.26)	1.10–2.29	3.8% (5.0)	0.902 (0.864–0.932)	2.8% (3.6)	0.929 (0.897–0.951)
Average C/D ratio	0.40 (0.15)	0.07–0.66	4.5% (8.4)	0.975 (0.964–0.983)	3.2% (4.8)	0.990 (0.985–0.993)
Vertical C/D ratio	0.39 (0.14)	0.06–0.63	7.5% (12)	0.942 (0.918–0.960)	4.6% (6.6)	0.977 (0.966–0.984)
Cup volume (mm^3^)	0.10 (0.10)	0.0–0.42	11.0% (23)	0.987 (0.982–0.991)	8.1% (12.4)	0.993 (0.990–0.995)

*C/D ratio* cup disc ratio, *CI* confidence interval, *CV* coefficient of variance, *ICC* intraclass correlation, *n* number of eyes

No statistically significant correlations were found between age and the optic disc parameters, including peripapillary RNFL thickness. Nor were any correlations found between refraction and the optic disc and RNFL. No statistically significant difference in terms of gender was found either.

A weak correlation between VA and average RNFL thickness (*r* = 0.24, *p* = 0.01) was found, but not with the other disc parameters.

**Fig. 1:**
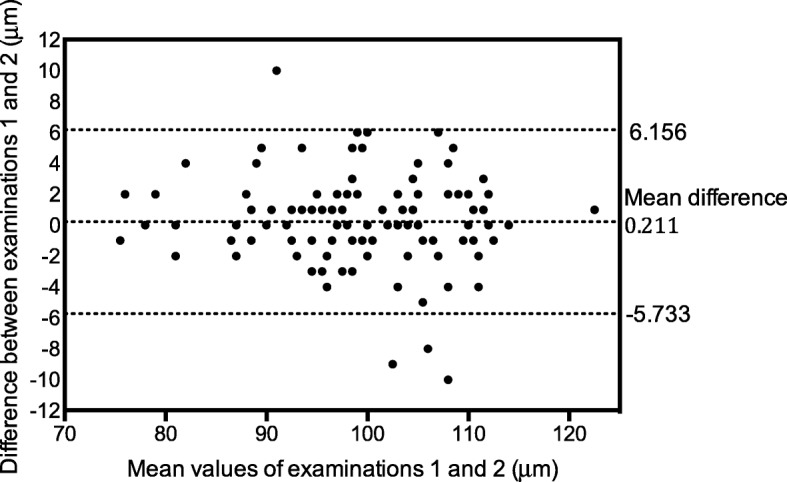
Bland–Altman plot of repeatability (intra-observer variability) in assessments of average RNFL thickness. The plot shows the differences between examinations 1 and 2

**Fig. 2:**
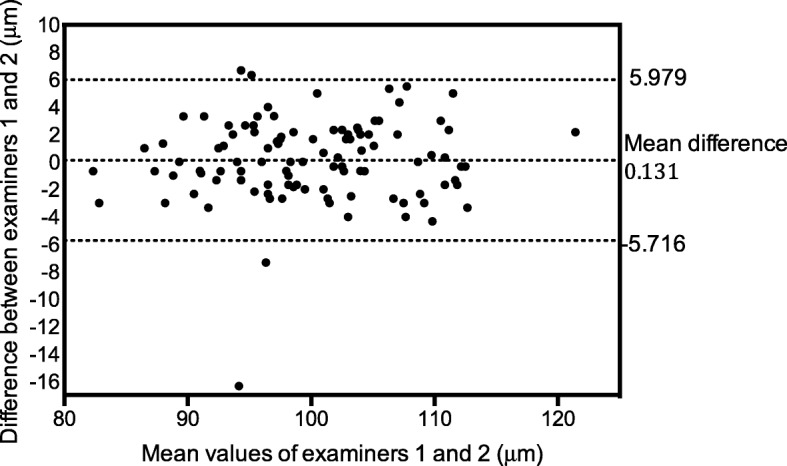
Bland–Altman plot of reproducibility (inter-observer variability) in assessments of average RNFL thickness. The plot shows the differences between examiners 1 and 2

Intra-observer measurement variability (repeatability) and inter-observer variability (reproducibility) expressed as CV and ICC, are presented in Table 1. In five eyes, assessed by the first examiner, only one of three examinations was performed and in two eyes the examination of the second examiner could not be performed. Consequently, the repeatability was analysed in 105 eyes and the reproducibility in 108 eyes. Figures 1 and 2 illustrate the repeatability and reproducibility of average RNFL thickness values as Bland–Altman plots.

In 53 children, both eyes were compared. Mean values for the right and left eyes in these children are given in Table 2, together with the correlation between the eyes and the interocular difference. Values from the right and left eyes correlated significantly (*p* < 0.01). There were no statistical differences between the eyes, except in RNFL thickness in the superior (p < 0.01), nasal (p < 0.01) and temporal (*p* < 0.05) sectors; see Table 2.

**Table 2:** Measures of symmetry between right and left in 53 pair of eyes assessed with Cirrus OCT

	Right eye	Left eye	Correlation RE-LE	Interocular difference RE-LE	
	Mean (SD)	Mean (SD)		Mean difference	95% limits of agreement
Average RFNL thickness (μm)	98.7 (8.4)	98.8 (8.9)	0.881**	− 0.126	−8.402–8.150
Inferior	130.1 (15.3)	129.7 (13.6)	0.706**	0.519	− 21.45–22.49
Superior	120.1 (14.1)	125.3 (14.7)	0.584**	− 5.226	−31.05–20.6
Nasal	75.8 (11.5)	72.9 (11.2)	0.734**	2.909	−13.39–19.21
Temporal	68.8 (8.7)	66.5 (7.6)	0.684**	2.230	−11.36–15.82
Disc area (mm^2^)	1.85 (0.39)	1.90 (0.32)	0.780**	− 0.044	−0.529–0.441
Rim area (mm^2^)	1.49 (0.26)	1.52 (0.26)	0.810**	− 0.035	−0.344–0.274
Average C/D ratio	0.39 (0.16)	0.41 (0.14)	0.989**	− 0.014	−0.210–0.181
Vertical C/D ratio	0.38 (0.16)	0.40 (0.13)	0.713**	− 0.022	−0.242–0.197
Cup volume (mm^3^)	0.10 (0.10)	0.09 (0.08)	0.832**	0.005	−0.106–0.117

**p < 0.01 *RE* right eye, *LE* left eye, *SD* standard deviation

## Discussion

In the present population-based study, we report normative data for the peripapillary RNFL thickness and optic disc parameters in healthy children aged 6–15. Repeatability (intra-observer) and reproducibility (inter-observer) were good overall, with high intraclass correlations and low coefficients of variances. There were no significant correlations with age, refraction or gender in this age group. The correlations between right and left eyes were good, and differences between the eyes were small.

Use of OCT has been widespread for assessments of the optic nerve, and knowledge of normal values in children is important. Different OCT devices can differ regarding the optic nerve parameters [14, 15]. In the present study, we used the Cirrus OCT, optic disc cube 200 × 200 protocol, as in the studies by Elia et al. [16], Altemir et al. [9, 17], Rao et al. [11], Barrio-Barrio et al. [5], Al-Haddad et al. [6] and, most recently, Pawar et al. [8] and Güragaç et al. [18]. The mean value of the RNFL thickness in our study (99 μm) resembled those found in the other studies, in which the average thickness was between 93 and 98 μm, the two Indian studies [8, 11] having the lower values (93–94 μm). It can be suspected that the lower values depend on ethnicity, since a difference in RNFL thickness among ethnic groups has been found in adults [19]. As in the other studies, the “ISNT rule” was followed, i.e. the RNFL was thickest in the inferior sector, followed by the superior, nasal and temporal sectors. The higher values in the superior and inferior sectors are probably caused by the larger number of fibres converging from the arcuate bundles into the optic nerve.

Regarding disc and rim sizes and cup/disc ratio our values resembled those found by Elia et al. [16], whereas Pawar et al. [8] reported larger disc size and smaller rim size and thus, a higher cup/disc ratio.

In adults, a thinning of the RNFL thickness with age has been reported [20]. However, the present study revealed no correlation between RNFL thickness and age, in accordance with other studies in children [5, 6, 8, 11, 16, 18]. The child’s age in this age range therefore need not be considered in interpretation of the results. Regarding refraction, no correlation was found in our study, in contrast to Al-Haddad et al. [6] who had a larger range of myopia and hypermetropia in their group and found a positive correlation between RNFL thickness and spherical equivalent. A positive correlation with refraction was also reported by Barrio-Barrio et al. [5], Güragaç et al. [18], and Rao et al. [11]. The lack of correlation in the present study might be explained by the narrow range of refractive error in the children. Finally, regarding gender, similar values were found in boys and girls, in accordance with the studies by Al-Haddad et al. [6], Barrio-Barrio et al. [5], Elia et al. [16] and Pawar et al. [8], but in contrast to the study by Rao et al. [11], who found thinner RNFL thickness in females.

Repeatability and reproducibility were good in the present study. Chung et al. [21] have compared adults and children using Spectralis OCT and found intra-observer variability to be almost as good in children as in adults. In a previous study, we found better repeatability regarding RNFL thickness using TD-OCT (Stratus) than when the Heidelberg Retina Tomograph (HRT) was used [22]. In this study, using SD-OCT, we found even better CVs and ICCs. In addition, regarding reproducibility the values were good, in accordance to one previous study in children using Cirrus-OCT [9]. The ICC was lowest in the temporal sector in the present study, in contrast to Altemir et al. who found the lowest ICC in the nasal sector. The differences between the studies could not be fully explained since both studies used Cirrus OCT and the same method [9]. Because of the good intra- and inter-observer correlations, we believe that measurements performed on different occasions and by different examiners, using Cirrus OCT, are reliable. One has to remember that the variability might be different in eyes with diseases. In adults, the effect of disease severity in glaucoma on reproducibility has been contradictory. Studies using Cirrus OCT on normal and glaucoma eyes have shown both similar [23] and worse [24] reproducibility in the affected eyes. Whether, the intra-and inter variations are affected in children with optic nerve diseases has to be further explored.

Knowledge of interocular differences in the measurements of normal eyes is important in evaluation of optic diseases. Regarding RNFL thickness and optic disc size, this has previously been reported in a few studies in children [8, 10, 11, 17]. The present study revealed a good correlation between RE and LE regarding the optic disc and RNFL thickness, and the mean differences were small; see Table 2. Statistically, however, there was a difference in the RNFL in the superior (thinner in REs), inferior and nasal (thinner in LEs) sectors, in accordance with Altemir et al. [17]. Interestingly, Al-Haddad et al. [10] and Pawar et al. [8] also found that the superior sector was thinner in the REs and the temporal sector in the LEs. In the superior sector in the present study, the interval of the 95% limits of agreement was − 31 to 20 μm. This must be considered in evaluation of monocular optic diseases.

### Strengths and limitations

The strength of the present study was that it was strictly population-based and that the same two experienced examiners performed all the examinations. The major limitation was the relatively small sample size, especially since the age range was rather broad. However, also in larger studies no correlation with age in this age span has been found. The narrow inclusion criteria of refractive error in the present study could also have been a bias. Finally, information of other morphometric data, such as ocular axial length and the child’s height and weight could have added further information to the study,

## Conclusion

In this population-based study normative data for optic nerve head and peripapillary RNFL thickness assessed with Cirrus OCT in 57 full-term, healthy children, aged 6–15 were reported. Repeatability (intra-observer) and reproducibility (inter-observer) were good. The interocular correlations were high although a difference between the right and left eyes in three sectors of the peripapillary RNFL was found. There were no correlations with age, refraction or gender in this age group.

## Abbreviations

C/D: Cup disc
CI: Confidence interval
CV: Coefficient of variance
D: Dioptres
HRT: Heidelberg retina tomograph
ICC: Intraclass correlation
ISNT: Inferior superior nasal temporal
LE: Left eye
N: Number
OCT: Optical coherence tomography
RE: Right eye
RNFL: Retinal nerve fibre layer
SD: Standard deviation
SD-OCT: Spectral domain optical coherence tomography
VA: Visual acuity

## References

[1] Hee MR, Izatt JA, Swanson EA, Huang D, Schuman JS, Lin CP, Puliafito CA, Fujimoto JG (1995). Optical coherence tomography of the human retina. Arch Ophthalmol.

[2] Eriksson U, Larsson E, Holmstrom G (2004). Optical coherence tomography in the diagnosis of juvenile X-linked retinoschisis. Acta Ophthalmol Scand.

[3] Holmstrom G, Eriksson U, Hellgren K, Larsson E (2010). Optical coherence tomography is helpful in the diagnosis of foveal hypoplasia. Acta Ophthalmol.

[4] Gospe SM, Bhatti MT, El-Dairi MA (2017). Emerging applications of optical coherence tomography in pediatric optic neuropathies. Semin Pediatr Neurol.

[5] Barrio-Barrio J, Noval S, Galdos M, Ruiz-Canela M, Bonet E, Capote M, Lopez M (2013). Multicenter Spanish study of spectral-domain optical coherence tomography in normal children. Acta Ophthalmol.

[6] Al-Haddad C, Barikian A, Jaroudi M, Massoud V, Tamim H, Noureddin B (2014). Spectral domain optical coherence tomography in children: normative data and biometric correlations. BMC Ophthalmol.

[7] Ayala Marcelo, Ntoula Evangelia (2016). Retinal Fibre Layer Thickness Measurement in Normal Paediatric Population in Sweden Using Optical Coherence Tomography. Journal of Ophthalmology.

[8] Pawar N, Maheshwari D, Ravindran M, Ramakrishnan R (2017). Interocular symmetry of retinal nerve fiber layer and optic nerve head parameters measured by cirrus high-definition optical tomography in a normal pediatric population. Indian J Ophthalmol.

[9] Altemir I, Pueyo V, Elia N, Polo V, Larrosa JM, Oros D (2013). Reproducibility of optical coherence tomography measurements in children. Am J Ophthalmol.

[10] Al-Haddad C, Antonios R, Tamim H, Noureddin B (2014). Interocular symmetry in retinal and optic nerve parameters in children as measured by spectral domain optical coherence tomography. Br J Ophthalmol.

[11] Rao A, Sahoo B, Kumar M, Varshney G, Kumar R (2013). Retinal nerve fiber layer thickness in children 5-18 years by spectral-domain optical coherence tomography. Semin Ophthalmol.

[12] Hedin A, Olsson K (1984). Letter legibility and the construction of a new visual acuity chart. Ophthalmologica.

[13] Bland JG, Altman DG (1986). Statistical methods for assessing agreement between two methods of clinical measurement. Lancet.

[14] Knight O, Chang R, Feuer W, Budenz D (2009). Comparison of retinal nerve fiber layer measurements using time domain and spectral domain optical coherence tomography. Ophthalmol.

[15] Giambene B, Virgili G, Menchini U (2017). Retinal nerve fiber layer thickness by stratus and cirrus OCT in retrobulbar optic neuritis and nonarteritic ischemic optic neuropathy. Eur J Ophthalmol.

[16] Elia N, Pueyo V, Altemir I, Oros D, Pablo L (2012). Normal reference ranges of optical coherence tomography parameters in childhood. Br J Ophthalmol.

[17] Altemir I, Oros D, Elia N, Polo V, Larrosa JM, Pueyo V (2013). Retinal asymmetry in children measured with optical coherence tomography. Am J Ophthalmol.

[18] Güragaç FB, Totan Y, Güler E, Tenlik A, Ertugrul IG (2017). Normaltive spectral domain optical coherence tomography data in healthy Turkish children. Semin Opthalmol.

[19] Knight O, Girkin C, Budenz D, Durbin M, Feuer W (2012). For the cirrus OCT normative database study group. Effect of race, age, and axial length on optic nerve head parameters and retinal nerve fiber layer thickness measured by cirrus HD-OCT. Arch Ophthalmol.

[20] Wu Z, Saunders L, Zangwill L, Daga F, Crowston J, Medeiros F (2017). Impact of normal aging and progression definitions on the specificity of detecting retinal nerve fiber layer thinning. Am J Ophthalmol.

[21] Chung HK, Han YK, Oh S, Kim SH (2016). Comparison of optical coherence tomography measurement reproducibility between children and adults. PLoS One.

[22] Larsson E, Eriksson U, Alm A (2011). Retinal nerve fibre layer thickness in full-term children assessed with Heidelberg retinal tomography and optical coherence tomography: normal values and interocular difference. Acta Ophthalmol.

[23] Vazirabi J, Kaushik S, Sing Pandav S, Gupta P (2015). Reproducability of retinal nerve fiber layer measurements acrss the glaucoma spectrum using optical coherence tomography. Indian J Ophthalmol.

[24] Suh MH, Yoo BW, Park KH, Kim H, Kim HC (2015). Reproducibility of spectral-domain optical coherence tomography RNFL map for glaucomatous and fellow normal eyes in unilateral glaucoma. J Glaucoma.

